# Two stage fracture of a polyethylene post in a 9-year-old posterior-stabilized knee prosthesis: a case report

**DOI:** 10.1186/1752-1947-4-65

**Published:** 2010-02-23

**Authors:** Fabio D'Angelo, Daniele Marcolli, Paolo Bulgheroni, Luigi Murena, Terenzio Congiu, Paolo Cherubino

**Affiliations:** 1 Department of Orthopaedics and Traumatology, University of Insubria, Ospedale di Circolo - Fondazione Macchi, V le Borri 57, 21100 Varese, Italy; 2 Department of Normal Human Morphology "L Cattaneo", University of Insubria, Via O Rossi 9, 21100 Varese, Italy

## Abstract

**Introduction:**

Several cases of tibial post breakage are reported in the literature. To the best of our knowledge, only three cases of NexGen knee prosthesis (Zimmer, Warsaw, Indiana, USA) tibial post failure have been reported.

**Case presentation:**

In November 1999, a 63-year-old Caucasian woman from Italy with a history of symptomatic left knee osteoarthritis underwent a total knee arthroplasty. In March 2008, while rising from a chair, she felt a sudden pain and instability in her left knee. She reported a fracture of the polyethylene post of the tibial insert. No malposition or malalignment of either the femoral or tibial components were identified. The polyethylene tibial insert was studied under light microscopy and scanning electron microscopy. The fracture was also noted to have occurred without any notable polyethylene wear.

**Conclusion:**

Scanning electron microscopy revealed two different damage patterns that could be explained with a two-stage rupture of our patient's polyethylene post. This could have been caused by a non-optimal ligamentous balancing during first implant surgery. Her knee probably developed a varus instability that weakened the post, and then a posterior anterior stress finally broke the polyethylene.

## Introduction

The interaction between the polyethylene post of the tibial tray and the femoral cam is necessary for the proper functioning of posterior stabilized (PS) knee prosthesis [1]. PS total knee arthroplasty (TKA) was developed to grant stability, to achieve a higher range of motion due to rollback, and to prevent posterior subluxation of the implant [2]. The polyethylene spine contacts the cam at approximately 70° of flexion, thus preventing posterior subluxation. Mediolateral stability, however, is dependent only on a well balanced and aligned knee [3].

Polyethylene wear is a complication that could contribute to aseptic loosening and osteolysis after TKA [4]. Acknowledged factors that can influence polyethylene wear include prosthesis design, manufacturing, and poor surgical technique [5,6].

Several cases of tibial post breakage are reported in the literature [7-13]. To the best of our knowledge, three cases of NexGen PS knee prosthesis (Zimmer, Warsaw, Indiana, USA) tibial post failure have been reported [14-16]. This case report focuses on light microscopy and scanning electron microscopy (SEM) evaluation of the broken polyethylene insert. This report also aims to explain a possible mechanism for the failure of tibial post.

## Case presentation

In November 1999, a 63-year-old Caucasian woman from Italy (weight = 100 kg, height = 1.60 m, body mass index = 39) with a history of symptomatic left knee osteoarthritis underwent a TKA in another hospital. The implant used was a NexGen PS knee prosthesis (Zimmer, Warsaw, Indiana, USA) with a tibial component size of 4, a femoral component size of D, and a polyethylene insert 10 mm in thickness. No problem was reported during the follow-up examination, and the patient was able to perform normal life activities for the next nine years.

**Figure 1:**
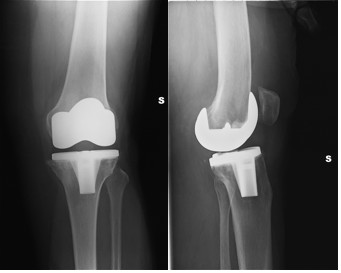
**Preoperative X-rays**. The hyperextension of the tibia in relation to the posterior subluxation of the femur can be seen.

In March 2008, while rising from a chair, she felt a sudden pain and instability in her left knee. After this acute event she was unable to bear weight on her left knee, and was thus forced to use crutches. On physical examination she presented a mild effusion of the knee, a flexion of 90°, and knee hyperextension. The joint presented signs of both anteroposterior and varus to valgus instability. X-ray examinations showing the anteroposterior view of the knee did not indicate any remarkable alteration in polyethylene wear, while the lateral view showed a hyperextension of the tibia with a posterior subluxation of the femur (Figure 1). The hypothesis of post breakage was thus made.

Our patient underwent diagnostic knee arthroscopy and was scheduled to have her polyethylene insert changed. The procedure granted a clear view of the polyethylene and the broken post in articulation. However, actual findings showed that the polyethylene had no relevant wear areas or alterations. Based on these findings, we performed an anterior approach with medial parapatellar arthrotomy. During surgery, samples of the periprosthetic tissue were taken in order to obtain a histological evaluation. These specimens were treated with haematoxylin-eosin and von Kossa staining, and were then studied under light microscopy using polarized light in order to detect the typical birefringence of the polyethylene debris.

The total knee components appeared to be well fixed intraoperatively. The polyethylene insert was substituted with a 12-mm CD LPS Flex articular surface (Zimmer, Warsaw, Indiana, USA). The stability in full extension, mid-flexion, and full flexion of the knee was tested intraoperatively, and appeared to be good. The patient had no postoperative complications and recovered well.

**Figure 2:**
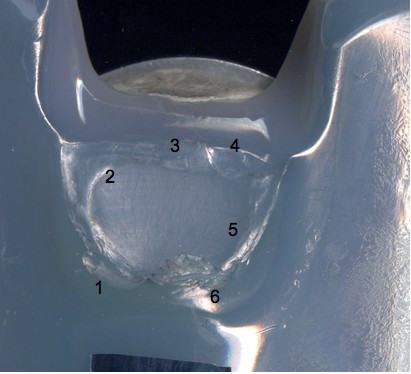
**Numerated areas of the polyethylene insert**.

The polyethylene insert and the broken post were both prepared for scanning electron microscopy (SEM) evaluation (Figure 2).

**Figure 3:**
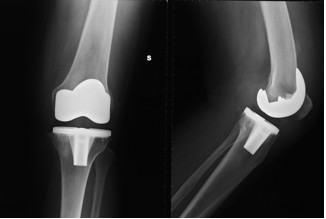
**X-rays at six months follow-up examination**.

At six months follow-up the patient had recovered complete function of her left knee. She was free from pain and could walk normally without any support (Figure 3).

## Discussion

The design feature common to all PS knee prosthesis is the cam-and-post mechanism that is incorporated into the femoral and tibial components. The cam on the femoral component is designed to engage the post of the tibial polyethylene during knee flexion. This interaction provides a functional substitute for the posterior cruciate ligament (PCL), thus resulting in femoral rollback as flexion increases. In addition, the cam and the post work to limit posterior displacement of the tibia relative to the femur in extension [1].

In some cases in which the resulting laxity in flexion is greater than the so-called "jump distance", or the height of the post, acute dislocation may occur. Another potential cause of flexion instability in a knee with PS prosthesis is the failure of the polyethylene post. This can be caused by either polyethylene acute fracture or fatigue fracture, which is a consequence of repetitive anterior impingement between the metal femoral cam and the polyethylene post [7].

No malposition or malalignment of both the femoral and tibial components were identified in our patient. Confirming the findings of Colizza *et al*., [17], polarized light microscopy did not reveal any notable polyethylene wear.

**Figure 4:**
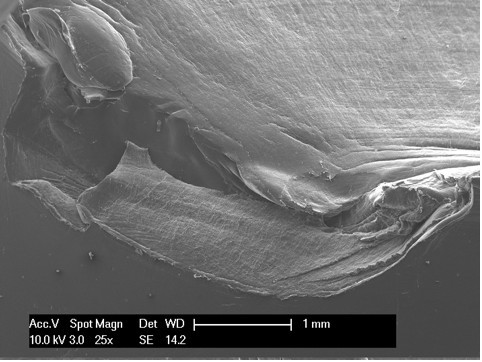
**Area 1 of Figure 2**.

**Figure 5:**
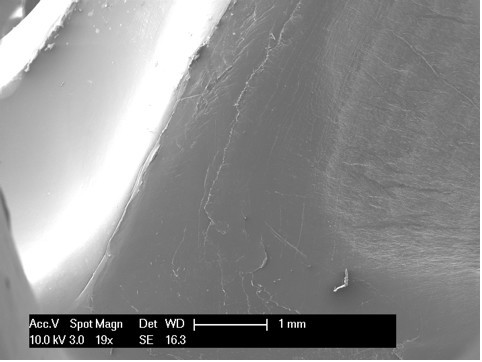
**Area 2 of Figure 2**.

**Figure 6:**
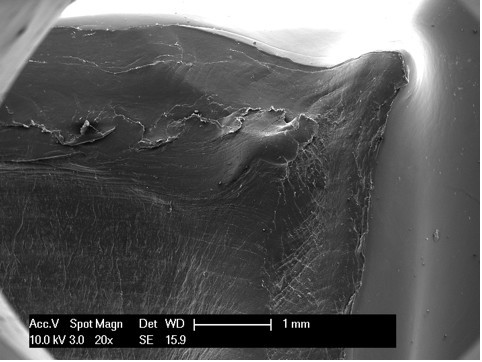
**Area 3 of Figure 2**.

Scanning electron microscopy, as reported in the literature [14,18], is an effective modality for analyzing the surface of fatigue fractures. An evaluation of the retrieved tibial polyethylene insert via SEM revealed two different damage patterns, considering the medial part and the lateral aspect (Figure 4). The medial part (Figure 2 areas 1, 2 and 3, Figure 4, Figure 5, Figure 6) presented a fracture line laminated in front and smooth behind and with the tear lines with a mediolateral and anterior posterior orientation.

The medial part of the fracture edge appears to be smooth (Figure 2 areas 2 and 3) and with a different orientation of the fracture lines. These characteristics suggest a chronic instability of the varus valgus knee prosthesis that slowly weakened the polyethylene post.

**Figure 7:**
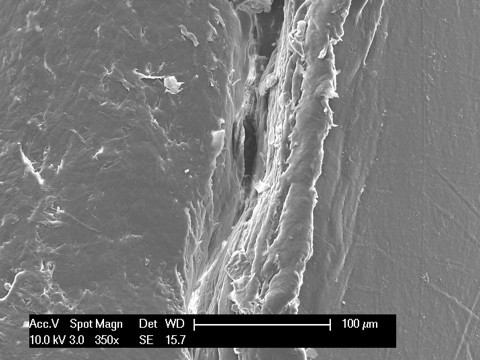
**Area 4 of Figure 2**.

**Figure 8:**
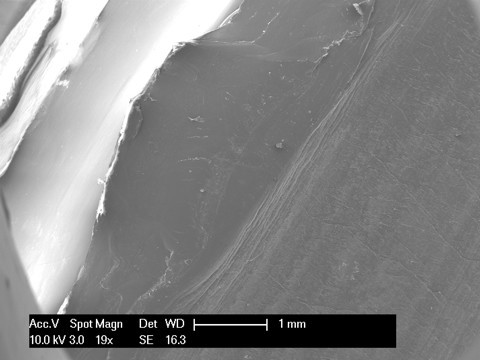
**Area 5 of Figure 2**.

**Figure 9:**
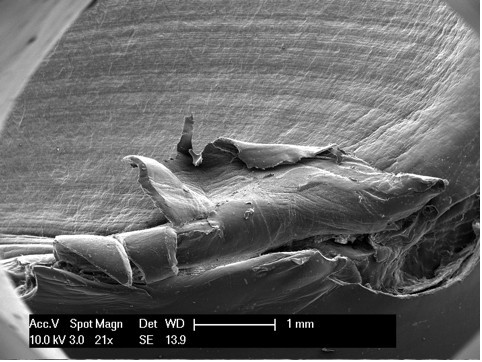
**Area 6 of Figure 2**.

Meanwhile, the lateral part (Figure 2 areas 4, 5 and 6, Figure 7, Figure 8, Figure 9) of the fracture presented a sharp line that ends anteriorly with a laminated tear (Figure 2 area 6, Figure 9) parallel to the anterior edge of the polyethylene insert. This implies that this area could be the terminal acute failure area of the fractured post. The final rupture occurred after the chronic weakening of the polyethylene due to the mediolateral stress on the tibial post. These features could be explained with a two-stage rupture of the polyethylene post. First, a varus and anterioposterior force caused partial rupture and instability of the post, which caused progressive smoothening of the medial and posterior fracture edges. Consequently, an anterioposterior lift-off force led to the complete rupture of the post. This could have been caused by a non-optimal ligamentous balancing during the first implant surgery. Our patient's knee probably developed progressive varus instability that slowly weakened the post, and then an anterioposterior stress finally broke the polyethylene.

**Figure 10:**
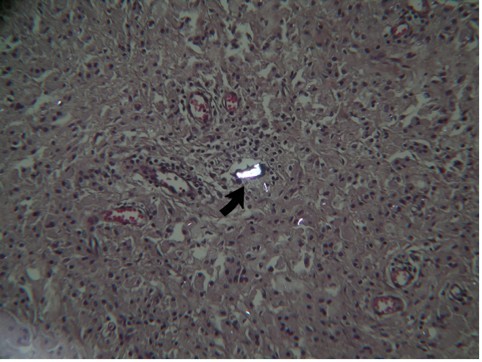
**Dense connective tissues with typical inflammatory cells and rare polyethylene flakes (arrow) are identifiable**.

Light microscopy evaluation showed a typical chronic inflammatory reaction. Rare polyethylene flakes were identifiable under polarized light. These particles appeared to be well-controlled by giant cells. No metallosis was observed (Figure 10). Von Kossa staining returned negative results. Such findings can be considered normal sinovia in TKA. The absence of polyethylene particles confirmed the macroscopic evidence of the absence of wear, which could have caused the breakage.

## Conclusion

Especially in posterior stabilized designs, it is important to achieve a well-balanced and aligned knee in order to reduce stress on the polyethylene spine that could otherwise lead to fatigue fracture [3].

We believe that the major failure mechanism of the polyethylene post in our patient was the mild varus valgus instability related to a non-optimal ligamentous balancing during her first implant surgery. This aspect, together with our patient's weight, produced a progressive weakening of the polyethylene post, which finally broke due to hyperextension mechanism.

Based on the experience of Callaghan *et al*. [19], proper femoral component positioning and avoiding excessive posterior tibial slope during implant surgery is crucial to reduce the anterior impingement of the post. Our patient's tibial slope was only 2°, which indicates a good compromise between ROM and tibial post impingement.

For most patients, once the diagnosis has been established the revision of the polyethylene insert is mandatory when components are well-fixed and in good alignment. In our patient, an insert that was only 2 mm thicker was enough to restore the stability of her knee. However, if the components are loose or malpositioned, complete revision surgery is recommended.

When sudden pain and instability appear in a functioning knee PS TKA, a tibial post breakage must be considered.

## Consent

Written informed consent was obtained from the patient for publication of this case report and accompanying images. A copy of the written consent is available for review by the Editor-in-Chief of this journal.

## Competing interests

The authors declare that they have no competing interests.

## Authors' contributions

FD performed the surgery, was involved in the bibliographic research, and was a major contributor in writing the manuscript. DM was involved in the bibliographic research and was also a major contributor in writing the manuscript. PB performed diagnostic knee arthroscopy. LM was involved in the bibliographic research. TC performed scanning electron microscopy evaluation and light microscopy of the samples from the patient. PC also performed surgery and contributed in writing the manuscript. All authors read and approved the final manuscript.
